# The Compound of Mangiferin-Berberine Salt Has Potent Activities in Modulating Lipid and Glucose Metabolisms in HepG2 Cells

**DOI:** 10.1155/2016/8753436

**Published:** 2016-03-30

**Authors:** Can Wang, Jian-Dong Jiang, Wei Wu, Wei-Jia Kong

**Affiliations:** ^1^Department of Virology, Institute of Medicinal Biotechnology, Chinese Academy of Medical Sciences and Peking Union Medical College, Beijing 100050, China; ^2^State Key Laboratory of Bioactive Natural Products and Function, Institute of Materia Medica, Chinese Academy of Medical Sciences and Peking Union Medical College, Beijing 100050, China; ^3^Changzhou Deze Medical Science Co., Ltd., Changzhou, Jiangsu 213100, China

## Abstract

The mangiferin-berberine (MB) salt was synthesized by ionic bonding of mangiferin (M) and berberine (B) at an equal molecular ratio. This study aimed to investigate the activities of MB salt in modulating lipid and glucose metabolisms in HepG2 cells. After 24 h treatment of the studying compounds, cellular AMP-activated protein kinase *α* (AMPK*α*)/acetyl-CoA carboxylase (ACC) protein levels and carnitine palmitoyltransferase (CPT) 1 activities, intracellular lipid contents, mRNA expression levels of target genes, glucose consumption, and glucose production amounts were determined. Compound C (CC) was used in the blocking experiments. Our results showed that MB salt increased p-AMPK*α* (Thr172)/p-ACC (Ser79) levels and CPT1 activity and suppressed oleic acid- (OA-) induced lipid accumulation and upregulation of lipogenic genes potently in HepG2 cells. The above activities of MB salt were AMPK dependent and were superior to those of M or B when administered at an equal molar concentration. MB salt enhanced basal and insulin-stimulated glucose consumption and suppressed gluconeogenesis more potently than M or B alone. The inhibiting activity of MB salt on cellular gluconeogenesis was AMPK dependent. Our results may support MB salt as a new kind of agent for the development of novel lipid or glucose-lowering drugs in the future.

## 1. Introduction

The metabolism syndrome (MS) is characterized by dyslipidemia, glucose intolerance and/or insulin resistance, hypertension, and obesity [1]. If there are no proper interventions, MS may lead to diabetes and complications, coronary heart disease, or even cancer eventually. Currently, chemical drugs such as biguanide and thiazolidinedione are commonly used in clinic for treatment of MS in order to improve metabolic disorders [1].

In addition to the abovementioned chemical drugs, numerous studies indicated that natural products isolated from plants might have beneficial effects in modulating lipid and glucose metabolisms, both* in vitro* and in animal models [2]. Some natural products are now subjected to clinical studies for the treatment of metabolic diseases; among them, a few compounds may have promising application prospects [3].

**Figure 1:**
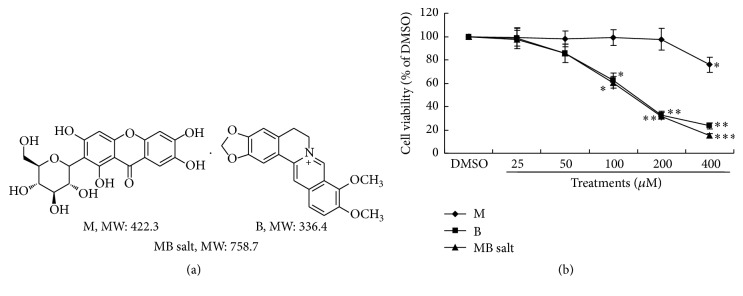
Influence of MB salt/M/B on cell viability. (a) Chemical structure and molecular weight (MW) of MB salt. (b) HepG2 cells were treated with the studying compounds at different concentrations for 24 h. Cell viability was determined by MTT staining and presented as percentages of control cells, which were treated with DMSO (0.5%) and defined as 100. Values are mean ± SD of 3 separate experiments; ^*∗*^ *p* < 0.05, ^*∗∗*^ *p* < 0.01, and ^*∗∗∗*^ *p* < 0.001 versus that of DMSO.

Mangiferin (M, Figure 1(a)), a xanthone glycoside, is a natural compound extracted from plants such as* Mangifera indica* and* Anemarrhena asphodeloides*. It was reported to have hypolipidemic [4–7], hypoglycemic [8–12], insulin-sensitizing [13], antiobesity [8, 9], antioxidative [14–16], and anti-inflammatory [14, 17] activities in animal models as well as in clinic. The beneficial effects of M on lipid and glucose metabolisms might be related to the activation of AMP-activated protein kinase (AMPK) [5, 17, 18], a key molecule that controls energy balance and metabolism in organisms [19].

Berberine (B, Figure 1(a)), an isoquinoline alkaloid, is a natural compound isolated from plants such as* Coptis chinensis*. B has a variety of pharmacological activities, and numerous studies demonstrated that it was a promising agent in modulating lipid and glucose metabolisms [3, 20, 21]. Now, B is undergoing clinical studies to systematically evaluate its efficacy and safety. The molecular mechanisms of B in modulating lipid and glucose metabolisms, which may include low-density lipoprotein receptor (LDLR) upregulation [22], AMPK activation [21], and gut microbiota modulation [23], are not fully elucidated and still need further investigation.

The compound of mangiferin-berberine (MB, Figure 1(a)) salt was synthesized by chemical bonding of M and B at an equal molecular ratio [24]. The nuclear magnetic resonance (NMR) data demonstrated that, in MB salt, M group (acidic) and B group (alkaline) conjugated by ionic bond to form a stable single molecule [24]. Due to the well-defined roles of M and B in improving metabolic disorders, it can be expected that this new compound may have favourable activities in modulating metabolism. Indeed, in pilot studies, the MB salt was found to stimulate the AMPK pathway in L6 skeletal muscle cells, lower blood glucose and lipids, and improve insulin sensitivity and liver function in KK-Ay diabetic mice [24]. However, the detailed activities and mechanisms of this new compound are still undefined. In the present report, we study the efficacies of MB salt in HepG2 cells and find that it has potent activities in stimulating AMPK and modulating lipid/glucose metabolisms, which are superior to those of M or B alone.

## 2. Materials and Methods

### 2.1. Chemicals and Reagents

The MB salt was synthesized by Changzhou Deze Medical Science Co., Ltd., as described previously [24]. M and B were supplied by the Northeast Pharmaceutical Group Shenyang No. 1 Pharmaceutical Co., Ltd. (Shenyang, China). Methylthiotetrazole (MTT), dimethyl sulphoxide (DMSO), compound C (CC), sodium L-lactate, oleic acid (OA), and bovine serum albumin (BSA) were purchased from Sigma-Aldrich Co. (St. Louis, MO, USA). Fetal bovine serum (FBS), Dulbecco's modified Eagle's medium (DMEM), glucose-, pyruvate-, and phenol red-free DMEM, sodium pyruvate, and Amplex Red Glucose/Glucose Oxidase Assay Kit were purchased from Gibco-Invitrogen (Grand Island, NY, USA). Reagents required for protein extraction and quantification were obtained from Thermo Fisher Scientific Inc. (Waltham, MA, USA) [25]. Monoclonal antibodies specific for AMPK*α*, phosphorylated AMPK*α* (p-AMPK*α*) (Thr172), acetyl-CoA carboxylase (ACC), phosphorylated ACC (p-ACC) (Ser79), and *β*-actin (ACTB) were obtained from Cell Signaling Technology, Inc. (Danvers, MA, USA). Immobilon®-P polyvinylidene difluoride (PVDF) membranes were from EMD Millipore Corporation (Billerica, MA, USA). Cell carnitine palmitoyltransferase (CPT) 1 Activity Assay Kit was from GenMed Scientifics Inc. (Shanghai, China). Steatosis Colorimetric Assay Kit was purchased from Cayman Chemical (Ann Arbor, MI, USA). Cell Triglyceride (TG) Assay Kit was from the Applygen Technologies Inc. (Beijing, China). Reagents required for RNA isolation, reverse transcription, and real-time PCR were purchased from Promega (Madison, WI, USA) [25]. Glucose Assay Kit (based on glucose oxidase method) was from Beijing Strong Biotechnologies, Inc. (Beijing, China). Human insulin (Humulin®) was purchased from Eli Lilly and Company (Shanghai, China).

### 2.2. Cell Culture

HepG2 cells were routinely cultured in DMEM plus 10% FBS and appropriate antibiotics in an atmosphere of 5% CO_2_ at 37°C. Before experiments, cells were trypsinized and allowed to grow to about 70–80% confluence. The cells were starved in 0.5% FBS-containing medium overnight before treatment.

### 2.3. Cell Viability Assay

HepG2 cells were seeded onto 96-well plates with 2 × 10^4^ cells per well. The studying compounds were dissolved with DMSO to make stock solutions at a concentration of 80 mM, which were stored at −20°C in aliquots. Before usage, the stock solutions were thawed and serially diluted with DMEM + 0.5% FBS. After 24 h of incubation and serum starvation, cells were treated with the studying compounds for 24 h as indicated (Figure 1(b)); each treatment had 5 replicate wells. Control cells were treated with 0.5% DMSO in DMEM + 0.5% FBS, a concentration with no cytotoxicity [26] and equal to the concentration of DMSO in cells treated with 400 *μ*M of studying compounds. Cell viability was determined by MTT staining as described previously [27]; the absorbance was read by a VICTOR*™* X4 Multilabel Plate Reader (PerkinElmer, Inc., Waltham, MA, USA) at a wavelength of 595 nm. The results were presented as percentages of control cells, which were defined as 100. The values of 50% inhibiting concentrations (IC_50_ values) of the compounds were calculated as described before [27].

### 2.4. Western Blot

After treatment with the studying compounds for 24 h, cell total proteins were extracted and quantified. Samples containing about 20 *μ*g of protein were used for 10% sodium dodecyl sulfate polyacrylamide gel electrophoresis (SDS-PAGE). The blots were then transferred from gels onto PVDF membranes as described previously [25]. After blocking, the protein levels of AMPK*α*, ACC, and ACTB were detected with specific monoclonal antibodies and an appropriate secondary antibody; signals were developed with an ECL kit (EMD Millipore Corporation). p-AMPK*α* (Thr172) and p-ACC (Ser79) levels were examined by phosphospecific antibodies after removal of antibody binding from the membranes. After scanning and quantification, the levels of p-AMPK*α* (Thr172) and p-ACC (Ser79) were normalized to those of AMPK*α* and ACC and plotted as indicated.

### 2.5. Cellular CPT1 Activity Assay

After treatment for 24 h, cells were harvested; samples containing 50 *μ*g of protein were used for CPT1 activity assay according to the supplier's protocol. The CPT1 experiments were repeated for 3 times; cellular CPT1 activities were presented as nmol of coenzyme A (CoA) produced in the assay system per minute.

### 2.6. Induction of Steatosis, Oil Red O (ORO) Staining, and Intracellular TG Assay

OA was dissolved in sterile phosphate buffered saline (PBS) + 5% BSA [28] to make a stock solution of 6 mM and was stored at −20°C in aliquots. Before usage, the stock solution was thawed and diluted with DMEM + 0.5% FBS for 10 times. HepG2 cells were seeded onto 6-well plates with 5 × 10^5^ cells per well. After 24 h of incubation and serum starvation, cells were left untreated or treated with 0.6 mM of OA for 24 h. MB salt/M/B were serially diluted from their stock solutions with DMEM + 0.5% FBS; at the same time of OA administration, they were added to the cells at indicated concentrations, except for some cells which were used as OA control. After treatment, intracellular lipids were stained with ORO by the Steatosis Colorimetric Assay Kit according to the supplier's protocol. After staining, the cells were observed under a light microscope and photographed. In parallel experiments, cells were harvested after treatment; intracellular TG contents were determined by the Cell TG Assay Kit and normalized to protein concentrations; mRNA levels of lipogenic transcription factors and their target genes (Table 1) were determined by real-time reverse transcriptase-polymerase chain reaction (RT-PCR).

### 2.7. RNA Extraction and Real-Time RT-PCR

Total RNA was isolated from cells and reversely transcribed into cDNAs according to the supplier's protocols. We performed real-time PCR in an ABI Prism® 7900 High-Throughput Real-Time PCR System (Applied Biosystems, Foster City, CA, USA) with gene specific primers (Table 1). The reaction condition was the same as our previous report [25]. For relative quantification of target genes, the comparative threshold cycle (C_T_) method was used with glyceraldehyde-3-phosphate dehydrogenase (GAPDH) as an internal control. The mRNA expression levels of target genes were plotted as fold of control cells, which were designated as 1.

### 2.8. Glucose Consumption Assay

HepG2 cells were seeded onto 24-well plates with 2 × 10^5^ cells per well. For basal glucose consumption assay, cells were treated with DMSO or the studying compounds for 24 h as indicated, with 4 replicate wells for each treatment. For insulin-stimulated glucose consumption assay, cells were treated with 0.05 nM of human insulin (diluted with DMEM + 0.5% FBS) together with DMSO or the studying compounds for 24 h. Glucose levels in the supernatant of media were assayed with a commercially available kit. Glucose consumption was calculated as glucose level of the fresh medium minus glucose level of the cultured medium.

### 2.9. Glucose Production Assay

Cells were seeded and treated as in the glucose consumption assay. After treatment with the studying compounds, cells were washed twice with PBS. The glucose production medium was prepared by adding sodium pyruvate and sodium L-lactate to the glucose-, pyruvate-, and phenol red-free DMEM to final concentrations of 2 mM and 20 mM, respectively. For one well of the 24-well plate, cells were loaded with 100 *μ*L of the glucose production medium and incubated for 4 h at 37°C. Glucose concentrations in the supernatant were analyzed by the Amplex Red Glucose/Glucose Oxidase Assay Kit according to the supplier's protocol. The values were normalized to protein concentrations and presented as percentages of DMSO control, which was defined as 100. In parallel experiments, cells were harvested for real-time RT-PCR analysis of the key genes involved in gluconeogenesis after 24 h treatment of studying compounds.

### 2.10. Blocking Experiments

After serum starvation, cells were pretreated with 10 *μ*M of CC (dissolved in DMSO) for 30 min; then studying compounds or OA were added. 24 h later, cells were harvested for western blot, CPT1 activity assay, oil red O (ORO) staining, intracellular TG assay, basal glucose consumption assay, glucose production assay, or real-time RT-PCR.

### 2.11. Statistical Analysis

Values are mean ± SD of 3-4 repeated experiments. After validation of the test for homogeneity of variance, differences among studying groups were examined by one-way ANOVA followed by the Newman-Keuls test for multiple comparisons. *p* < 0.05 was considered to be statistically significant.

## 3. Results

### 3.1. Cytotoxicities of Studying Compounds

First, we determined the influences of MB salt/M/B on cell viability by the MTT method. As shown in Figure 1(b), after 24 h treatment, M alone reduced the viability of HepG2 cells only when its concentration reached 400 *μ*M (*p* < 0.05 versus DMSO). The IC_50_ of M is larger than 400 *μ*M. For B and MB salt, cell viabilities declined slightly with no statistical significance when their concentrations reached 50 *μ*M, which was in agreement with a previous report [29]. When the concentrations of B and MB salt reached 100 *μ*M, cell viabilities declined significantly as compared to that of DMSO (*p* < 0.05). And when their concentrations reached 400 *μ*M, there were only averagely 23.4% and 15.3% living cells left after 24 h treatment (*p* < 0.01 or *p* < 0.001 versus DMSO). The IC_50_ values of B and MB salt were 133.9 ± 10.6 *μ*M and 131.0 ± 9.4 *μ*M, respectively. Our result was close to a previous report, in which the IC_50_ value of B was 42.33 *μ*g/mL in HepG2 cells [30]. We observed that the cells remained in good and healthy state if the concentrations of B and MB salt did not exceed 50 *μ*M. So, the maximum concentrations of the studying compounds we used in the following experiments were 50 *μ*M.

### 3.2. MB Salt Activates AMPK More Potently Than M or B Alone

**Figure 2:**
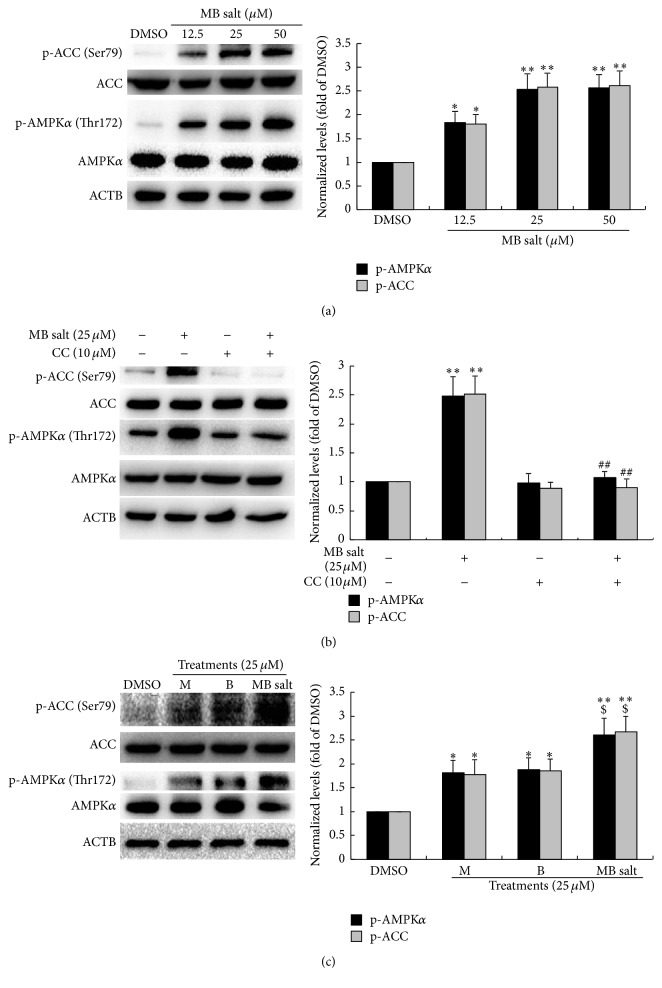
Stimulating effect of MB salt on the AMPK pathway. After serum starvation, cells were treated with different concentrations of MB salt for 24 h (a). Alternatively, cells were pretreated with CC for 30 min; then MB salt was added and incubated for 24 h (b). In the comparison experiment, an equal concentration of MB salt/M/B was used to treat the cells for 24 h (c). DMSO (0.1%) was used as control. After treatment, cell total proteins were extracted; the levels of p-AMPK*α* (Thr172), AMPK*α*, p-ACC (Ser79), ACC, and ACTB were determined by western blot. Representative blots are presented. The protein levels of p-AMPK*α* (Thr172) and p-ACC (Ser79) were normalized to those of AMPK*α* and ACC, respectively, and plotted as fold of DMSO treated cells. Values are mean ± SD of 3 separate experiments; ^*∗*^ *p* < 0.05 and ^*∗∗*^ *p* < 0.01 versus that of DMSO; ^##^ *p* < 0.01 versus that of MB salt alone in (b); ^$^ *p* < 0.05 versus those of M alone or B alone in (c).

As shown in Figure 2(a), MB salt increased the levels of p-AMPK*α* (Thr172) and p-ACC (Ser79) in dose-dependent manners after 24 h of administration. MB salt at 12.5 *μ*M could stimulate the cellular AMPK pathway significantly (*p* < 0.05 versus DMSO). The stimulating activities of MB salt on the phosphorylation of AMPK and ACC were completely blocked by CC (Figure 2(b)), a specific inhibitor of AMPK. To compare the bioactivities of MB salt/M/B in stimulating AMPK, we used these compounds to treat HepG2 cells at an equal molar concentration. As shown in Figure 2(c), when administered alone, 25 *μ*M of M and B could increase p-AMPK*α* (Thr172) and p-ACC (Ser79) levels by about 78%–85% (*p* < 0.05 versus DMSO). For comparison, MB salt at 25 *μ*M increased p-AMPK*α* (Thr172) and p-ACC (Ser79) levels averagely by 1.61- and 1.67-fold, respectively (*p* < 0.01 versus DMSO). The efficacy of MB salt on the AMPK/ACC pathway was significantly superior to that of M or B alone (*p* < 0.05).

**Figure 3:**
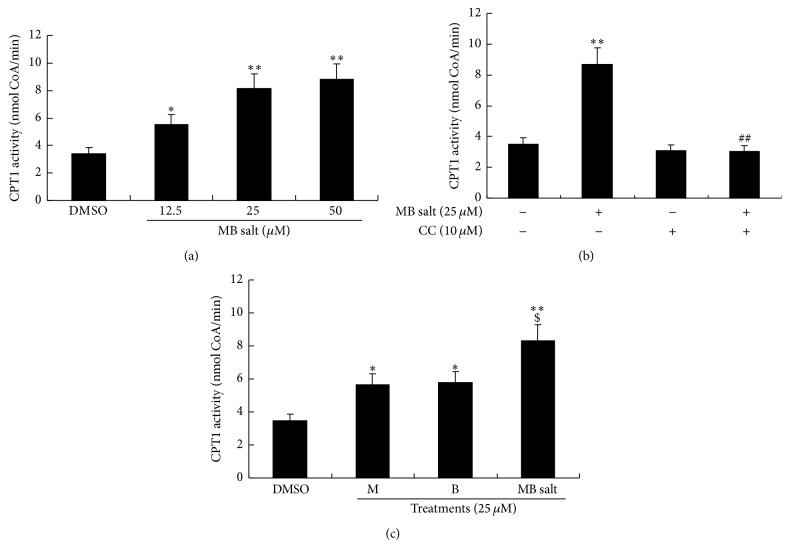
Stimulating effect of MB salt on CPT1 activity. HepG2 cells were treated the same as Figure 2. Cellular CPT1 activities were determined by a commercially available kit using equal amounts of proteins. Values are mean ± SD of 3 separate experiments; ^*∗*^ *p* < 0.05 and ^*∗∗*^ *p* < 0.01 versus that of DMSO; ^##^ *p* < 0.01 versus that of MB salt alone in (b); ^$^ *p* < 0.05 versus that of M alone or B alone in (c).

We then determined the influences of the studying compounds on cellular CPT1 activity. As shown in Figure 3(a), in HepG2 cells, MB salt enhanced CPT1 activity in a manner similar to that of AMPK, which could be abolished by CC pretreatment (Figure 3(b)). And again, the activity of MB salt on CPT1 was stronger than that of M or B when treated alone (*p* < 0.05) (Figure 3(c)). Taken together, the above results demonstrate that MB salt is a potent AMPK activator and its bioactivity is superior to that of M or B alone.

### 3.3. MB Salt Suppresses Hepatic Steatosis More Effectively Than M or B Alone

**Figure 4:**
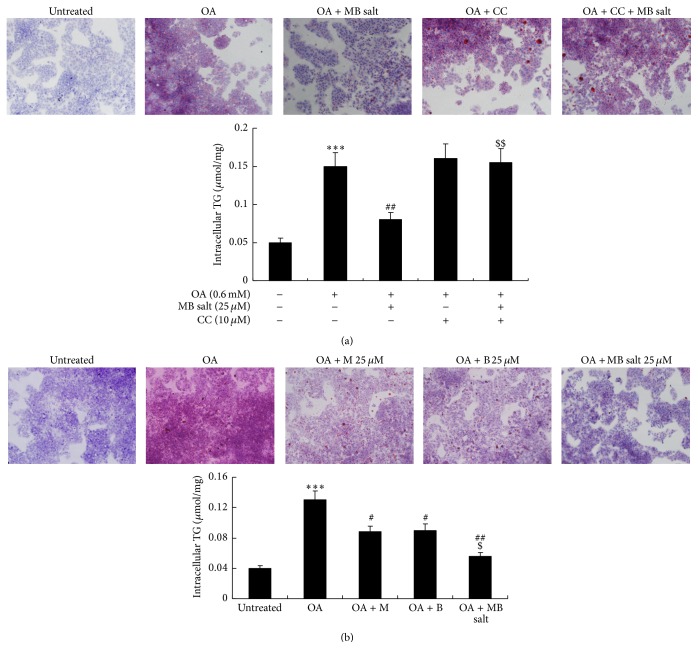
Effects of MB salt on OA-induced steatosis and TG accumulation. (a) HepG2 cells were left untreated or pretreated with CC for 30 min; then the cells were again left untreated or treated with OA or OA + MB salt as indicated. 24 h later, cells were used for ORO staining (upper panel) or harvested for intracellular TG assay (lower panel). Representative pictures are presented. Values are mean ± SD of 4 separate experiments; ^*∗∗∗*^ *p* < 0.001 versus that of untreated cells; ^##^ *p* < 0.01 versus that of OA alone; ^$$^ *p* < 0.01 versus that of OA + MB salt. (b) Cells were left untreated or treated with OA and an equal concentration of MB salt/M/B as indicated for 24 h. Then, cells were used for ORO staining (upper panel) or intracellular TG assay (lower panel). Representative pictures are presented. Values are mean ± SD of 4 separate experiments; ^*∗∗∗*^ *p* < 0.001 versus that of untreated cells; ^#^ *p* < 0.05 and ^##^ *p* < 0.01 versus that of OA alone; ^$^ *p* < 0.05 versus that of OA + M or OA + B.

To investigate the influence of MB salt on lipid metabolism, HepG2 cells were challenged with OA [31]. As shown in Figure 4, 0.6 mM of OA treatment for 24 h induced steatosis and increased intracellular TG level (*p* < 0.001 versus untreated) dramatically in HepG2 cells, as determined by ORO staining and intracellular TG assay. Coadministration of MB salt prevented steatosis and reduced intracellular TG content in dose-dependent manners (data not shown). The efficacies of MB salt were completely blocked by CC pretreatment (Figure 4(a)). The MB salt suppressed hepatic steatosis and TG accumulation more effectively than M or B when administered at an equal molar concentration. As shown in Figure 4(b), 25 *μ*M of MB salt treatment for 24 h reduced intracellular TG content averagely by 56.9% (*p* < 0.01 versus OA alone), which was superior to that of M or B alone (*p* < 0.05).

**Figure 5:**
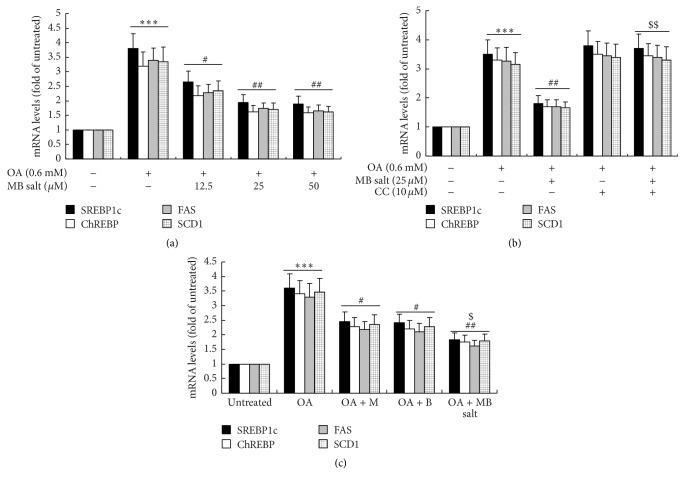
Effects of MB salt on the expression levels of lipogenic transcription factors and their target genes. (a) HepG2 cells were left untreated or treated with OA or OA + MB salt as indicated. ^*∗∗∗*^ *p* < 0.001 versus that of untreated cells; ^#^ *p* < 0.05 and ^##^ *p* < 0.01 versus that of OA alone. (b) Cells were treated as described in Figure 4(a). ^*∗∗∗*^ *p* < 0.001 versus that of untreated cells; ^##^ *p* < 0.01 versus that of OA alone; ^$$^ *p* < 0.01 versus that of OA + MB salt. (c) Cells were treated as described in Figure 4(b). ^*∗∗∗*^ *p* < 0.001 versus that of untreated cells; ^#^ *p* < 0.05 and ^##^ *p* < 0.01 versus that of OA alone; ^$^ *p* < 0.05 versus that of OA + M or OA + B. After treatment for 24 h, cell total RNA was extracted for real-time RT-PCR determination of mRNA levels of indicated genes, which were normalized to that of GAPDH and plotted as fold of untreated cells. Values are mean ± SD of 3 separate experiments.

In parallel with the development of steatosis, the mRNA expression levels of lipogenic transcription factors like sterol regulatory element-binding protein 1c (SREBP1c) and carbohydrate responsive element-binding protein (ChREBP) as well as their target genes like fatty acid synthase (FAS) and stearoyl-CoA desaturase 1 (SCD1) increased greatly after OA administration (Figure 5, *p* < 0.001 versus untreated). The MB salt suppressed the upregulation of the above genes in dose-dependent (Figure 5(a)) and AMPK-dependent (Figure 5(b)) manners. In agreement with its stimulating activity on AMPK and inhibiting activity on steatosis, the MB salt reduced the expression levels of lipogenic genes more effectively than M or B alone (*p* < 0.05, Figure 5(c)). Taken together, these results indicate that MB salt suppresses lipogenesis, steatosis, and TG accumulation in HpeG2 cells through AMPK activation, and its efficacies are more potent than M or B alone.

**Figure 6:**
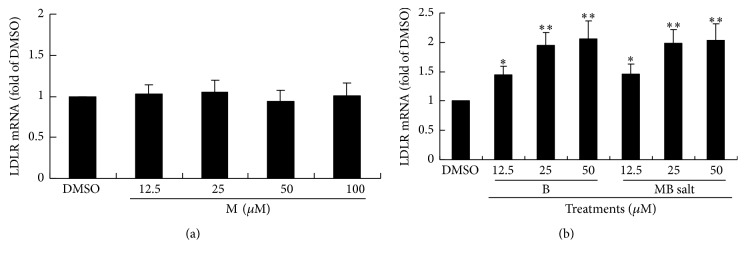
Effects of MB salt/M/B on LDLR mRNA levels. HepG2 cells were treated with different concentrations of M (a), B, or MB salt (b) for 24 h. DMSO (0.1%) was used as control. Cell total RNA was extracted for real-time RT-PCR determination of LDLR mRNA levels, which were normalized to that of GAPDH and plotted as fold of DMSO. Values are mean ± SD of 3 separate experiments; ^*∗*^ *p* < 0.05 and ^*∗∗*^ *p* < 0.01 versus that of DMSO.

As B is able to upregulate LDLR mRNA expression in liver cells [22], we want to know whether or not M has the same activity. As shown in Figure 6(a), M alone had no influence on hepatic LDLR expression even when its concentration reached 100 *μ*M. On the contrary, LDLR mRNA level could be upregulated by B at 12.5 *μ*M in HepG2 cells (*p* < 0.05 versus DMSO) (Figure 6(b)). The activity of MB salt on LDLR expression was the same as that of B alone (Figure 6(b)).

### 3.4. MB Salt Stimulates Glucose Consumption and Suppresses Gluconeogenesis More Potently Than M or B Alone

**Figure 7:**
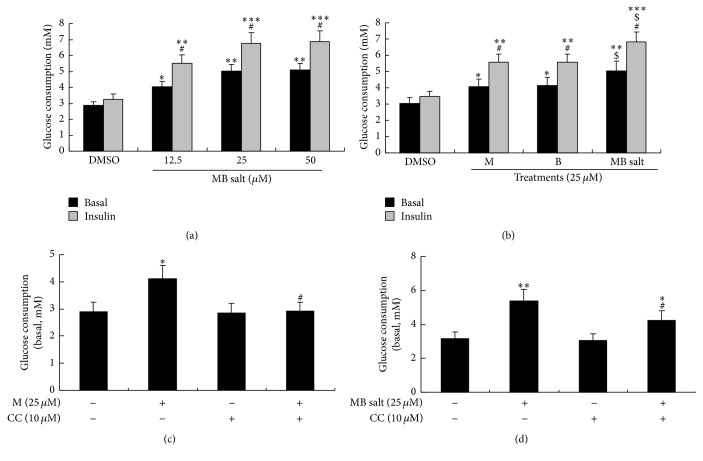
Effects of MB salt/M/B on basal and insulin-stimulated glucose consumptions. (a) After serum starvation, cells were treated with DMSO (0.1%) or different concentrations of MB salt with or without 0.05 nM of human insulin for 24 h. Glucose levels in the culture media were assayed and glucose consumptions were calculated as described in Section 2. Values are mean ± SD of 3 separate experiments; ^*∗*^ *p* < 0.05, ^*∗∗*^ *p* < 0.01, and ^*∗∗∗*^ *p* < 0.001 versus that of DMSO; ^#^ *p* < 0.05 versus that of basal glucose consumption. (b) HepG2 cells were treated with DMSO (0.1%) or an equal concentration of MB salt/M/B with or without 0.05 nM of human insulin for 24 h; glucose consumptions were then calculated. Values are mean ± SD of 3 separate experiments; ^*∗*^ *p* < 0.05, ^*∗∗*^ *p* < 0.01, and ^*∗∗∗*^ *p* < 0.001 versus that of DMSO; ^#^ *p* < 0.05 versus that of basal glucose consumption; ^$^ *p* < 0.05 versus that of M alone or B alone. (c and d) Cells were pretreated with CC for 30 min; then M (c) or MB salt (d) was added and incubated for 24 h. DMSO (0.1%) was used as control. After treatment, basal glucose consumptions were calculated. Values are mean ± SD of 3 separate experiments; ^*∗*^ *p* < 0.05 and ^*∗∗*^ *p* < 0.01 versus that of DMSO; ^#^ *p* < 0.05 versus that of M alone (c) or MB salt alone (d).

Next, we investigated the activities of the studying compounds on glucose metabolism. As shown in Figure 7(a), MB salt stimulated basal glucose consumption in HepG2 cells in a dose-dependent manner. The efficacy of MB salt was superior to that of M or B (*p* < 0.05) when administered at an equal molar concentration (Figure 7(b)). Furthermore, our result showed that the basal glucose consumption-stimulating activity of B was not influenced by CC (data not shown), which was in agreement with a previous report [29]. On the contrary, pretreatment of CC totally abolished the stimulating activity of M on cellular basal glucose consumption (Figure 7(c)), indicating that the activity of M was AMPK dependent. Interestingly, when MB salt was used to treat the cells together with CC, its activity on basal glucose consumption was partially inhibited. As shown in Figure 7(d), cellular basal glucose consumption in the presence of MB salt + CC was significantly less than that of MB salt alone (*p* < 0.05), but still higher than DMSO treated cells (*p* < 0.05). This result was consistent with the blocking experiments using M (Figure 7(c)) or B (data not shown) alone.

The above results indicated that MB salt itself could stimulate glucose metabolism without insulin. Previous studies by us and other researchers showed that M and B could stimulate insulin sensitivity [13, 20]. So, we also determined the influences of these compounds on insulin-stimulated glucose consumption in HpG2 cells. As shown in Figures 7(a) and 7(b), 0.05 nM of human insulin treatment for 24 h caused a slight increase of glucose consumption with no statistical significance. The MB salt enhanced insulin-stimulated glucose consumption dose-dependently (Figure 7(a)) and more potently than M or B alone (*p* < 0.05, Figure 7(b)). Notably, when MB salt/M/B were coadministered with 0.05 nM of insulin, additional glucose consumptions could be obtained as compared to those of basal glucose consumptions (*p* < 0.05, Figures 7(a) and 7(b)). Taken together, these results suggest that MB salt has potent activity in stimulating both basal and insulin-stimulated glucose consumptions in HpeG2 cells and its activities are superior to those of M or B.

**Figure 8:**
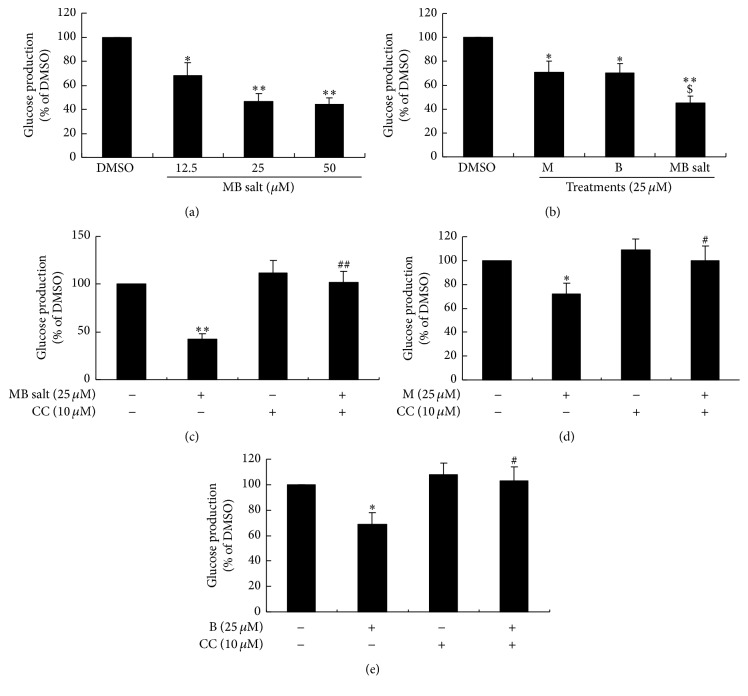
Effects of MB salt/M/B on glucose production. HepG2 cells were treated with different concentrations of MB salt (a) or an equal concentration of MB salt/M/B (b) for 24 h. Alternatively, cells were pretreated with CC for 30 min; then MB salt (c), M (d), or B (e) was added and incubated for 24 h. DMSO (0.1%) was used as control. After treatment, culture media were discarded; cells were loaded with the glucose production medium as described in Section 2. Four hours later, glucose levels in the supernatant were determined, normalized to protein concentrations, and presented as percentages of DMSO. Values are mean ± SD of 3 separate experiments; ^*∗*^ *p* < 0.05 and ^*∗∗*^ *p* < 0.01 versus that of DMSO; ^$^ *p* < 0.05 versus that of M alone or B alone in (b); ^#^ *p* < 0.05 and ^##^ *p* < 0.01 versus that of MB salt alone (c), M alone (d), or B alone (e).

**Figure 9:**
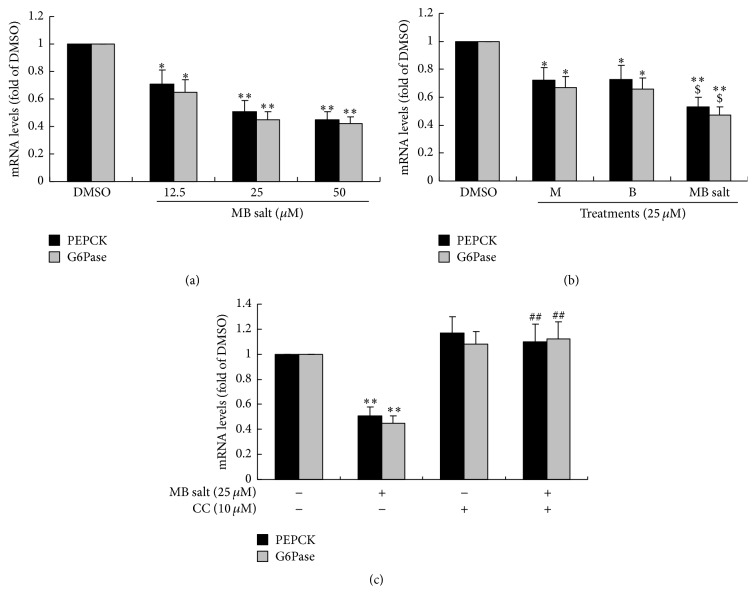
Effects of MB salt/M/B on PEPCK/G6Pase mRNA levels. Cells were treated as in Figure 8. After 24 h of treatment, cell total RNA was extracted for real-time RT-PCR determination of PEPCK/G6Pase mRNA levels, which were normalized to that of GAPDH and plotted as fold of DMSO. Values are mean ± SD of 3 separate experiments; ^*∗*^ *p* < 0.05 and ^*∗∗*^ *p* < 0.01 versus that of DMSO; ^$^ *p* < 0.05 versus those of M alone or B alone in (b); ^##^ *p* < 0.01 versus that of MB salt alone in (c).

AMPK activation was proved to inhibit gluconeogenesis [19], so we also determined the influences of these compounds on gluconeogenesis. As shown in Figure 8(a), MB salt inhibited glucose production dose-dependently in HepG2 cells. Accordingly, the expression levels of phosphoenolpyruvate carboxykinase (PEPCK)/glucose-6-phosphatase (G6Pase), two key enzymes of the gluconeogenesis pathway, were greatly downregulated by MB salt (Figure 9(a)). When administered at an equal molar concentration, MB salt had stronger activities in inhibiting glucose production (Figure 8(b)) and downregulating PEPCK/G6Pase (Figure 9(b)) than M or B alone (*p* < 0.05). Furthermore, unlike glucose consumption, the suppressing activities of MB salt/M/B on glucose production (Figures 8(c), 8(d), and 8(e)) and PEPCK/G6Pase expression (Figure 9(c) and data not shown) were totally abolished by CC. These results prove that MB salt suppresses gluconeogenesis in HepG2 cells through AMPK activation, and its efficacies are more potent than M or B alone.

## 4. Discussion

Here we report for the first time that MB salt, a novel compound synthesized by conjugation of natural products M and B, is a potent AMPK activator and has strong activities in modulating lipid and glucose metabolisms in HepG2 cells. As a single molecule, the advantages of M and B are able to be combined in MB salt, which has greater effectiveness in modulating lipid and glucose metabolisms as compared to either agent alone.

**Figure 10:**
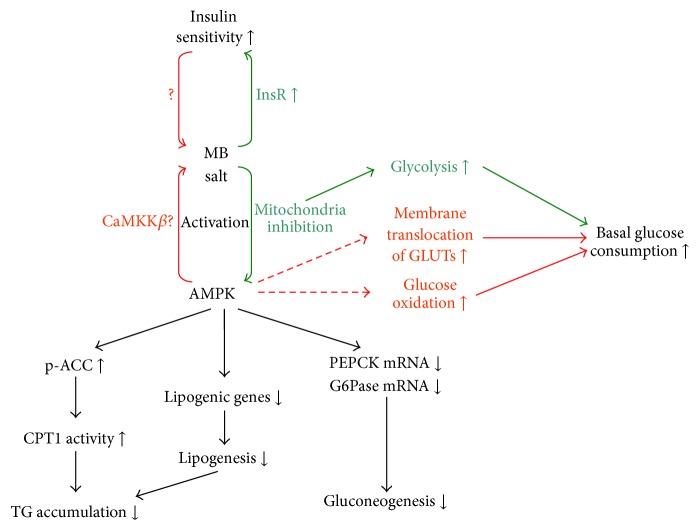
A schematic figure summarizes the activities and possible pathways of MB salt in modulating lipid and glucose metabolisms. In HepG2 cells, MB salt could activate AMPK potently, during which M (in red lines) and B (in green lines) might have different modes of actions. AMPK activation could cause the phosphorylation and inhibition of ACC, which in turn stimulated cellular CPT1 activity and suppressed TG accumulation. On the other hand, AMPK activation could result in the downregulation of lipogenic genes, which could reduce intracellular TG as well. PEPCK and G6Pase mRNA expression levels cloud also be downregulated by AMPK activation, which could cause inhibition of gluconeogenesis. M and B might enhance basal glucose consumption through different mechanisms. While B was proved to increase glycolysis and basal glucose consumption in an AMPK-independent manner, the activity of M on basal glucose consumption was AMPK dependent. M was shown to stimulate glucose metabolism by increasing membrane translocation of GLUTs and enhancing glucose oxidation, two processes that were able to be modulated by AMPK. In addition, MB salt could enhance insulin sensitivity potently, although M and B might act through different mechanisms. Dashed lines and question marks represent pathways and mechanisms that need further investigation and validation.

The activities and possible cellular pathways of MB salt in modulating lipid and glucose metabolisms are summarized in Figure 10. Our results revealed that MB salt could activate the AMPK pathway to a great extent in HepG2 cells at basal state. In another study, we proved that when treated with OA, the level of p-AMPK*α* (Thr172) was downregulated in liver cells [32], which was in accordance with the increase of intracellular TG (Figure 4) and the upregulation of lipogenic genes (Figure 5). Our results suggested that the MB salt should also stimulate the AMPK pathway in OA-treated HepG2 cells, as blocking AMPK with CC totally abolished its suppressing efficacies on intracellular TG accumulation and lipogenic gene upregulation induced by OA.

As AMPK activators, M and B might act through different mechanisms. It was reported that B could inhibit mitochondrial respiratory chain complex I in liver cells and skeletal muscle cells, which resulted in the reduction of ATP biosynthesis and subsequent increase of AMP/ATP ratio [29, 33]. Currently, it is generally accepted that B stimulates AMPK by increasing the cellular AMP/ATP ratio [29, 33, 34].

On the other hand, how AMPK was activated by M was not fully elucidated yet. One report suggested that M could also increase AMP/ATP ratio [5]. According to that report [5], M might stimulate cellular AMPK through a mechanism similar to that of B. However, another study demonstrated that M in fact enhanced oxygen consumption and ATP production but suppressed anaerobic respiration in muscle cells [9]. Interestingly, a recent study also showed that M had no influence on ATP amounts in 3T3-L1 preadipocytes [8]. In that study [8], the stimulating effect of M on AMPK was independent of liver kinase B1 (LKB1) but could be partially blocked by an inhibitor of calcium/calmodulin-dependent protein kinase kinase *β* (CaMKK*β*), an upstream kinase of AMPK. These findings suggest that the detailed mechanisms used by M to stimulate AMPK, which may involve CaMKK*β*, still need further investigation.

Although M and B might stimulate AMPK through different pathways, their activities were seemingly additive in the compound of MB salt (Figure 2(c)). AMPK is able to phosphorylate and suppress the catalytic activity of ACC [19]. The catalysate of ACC, malonyl-CoA, is an inhibitor of CPT1. AMPK activation will stimulate CPT1, which lead to the enhancement of fatty acid *β*-oxidation in the mitochondria [19]. Our results proved that MB salt stimulated CPT1 activity more effectively than M or B alone, which was in agreement with the cellular AMPK activity. As a result, OA-induced steatosis and TG accumulation in HepG2 cells were prevented by MB salt greatly, which might be due to the enhancement of fat burning. On the other hand, AMPK activation could inhibit lipogenesis by downregulating key lipogenic genes, as demonstrated by our results and other reports [19, 35]. The attenuation of lipogenesis by MB salt might also contribute to its suppressing efficacy on hepatic steatosis (Figure 10).

Our results showed that M had no effect on LDLR expression in HepG2 cells and that the LDLR-upregulating activity of MB salt was identical to that of B. Although M was efficacious in lowering serum TG in animal models [4–6] and in a clinical study [7], its activity on cholesterol was controversial. For example, while some reports suggested that M could reduce serum cholesterol and low-density lipoprotein cholesterol (LDL-c) levels [6, 36], other reports showed that it had no influence on serum or hepatic cholesterol levels [4, 5, 7]. The influence of M on cholesterol metabolism needs further investigation. We infer that M may have beneficial effects on cholesterol metabolism, but in a LDLR-independent manner.

M and B suppressed glucose production and PEPCK/G6Pase expression through AMPK activation, which suggested that they negatively regulated gluconeogenesis through a common cellular pathway. Accordingly, MB salt suppressed cellular gluconeogenesis more potently than M or B alone.

Unlike that of gluconeogenesis, M and B increased basal glucose consumption through different mechanisms (Figure 10). There were evidences indicating that in liver and muscle cells B stimulated basal glucose consumption in an AMPK-independent manner [29] and that it promoted glucose metabolism through induction of glycolysis [29, 34], which might be due to ATP inhibition by this compound [29, 33, 34]. On the contrary, M enhanced basal glucose consumption in an AMPK-dependent manner in our experiments. M was shown to stimulate glucose metabolism by increasing glucose oxidation and ATP production in muscle cells [9]. In addition, M might also stimulate the membrane translocation of glucose transporters (GLUTs) [8, 37], which could result in an increase of glucose uptake. It should be noted that glucose oxidation and the membrane translocation of GLUTs were able to be modulated by AMPK [3, 38]. Whether M stimulates these processes through the AMPK pathway needs further investigation. The influence of B on GLUTs was controversial and some researchers considered that modulation of GLUTs might not be the major mechanism of B to stimulate glucose utilization [21].

Our results proved that all three studying compounds could enhance the insulin-stimulated glucose consumption in HpeG2 cells. When coadministered with insulin, the glucose consumption-stimulating efficacies of MB salt/M/B were significantly increased. Considering the low concentration of insulin used in our experiment, it could be inferred that MB salt/M/B revealed insulin-sensitizing effects. The detailed mechanisms of M and B to increase insulin sensitivity are not fully elucidated. Our previous results showed that B could enhance insulin signaling through increasing the expression of insulin receptor (InsR) in liver cells [39]. M had no influence on the Akt pathway [18, 37] but was shown to induce CD36 redistribution [13] and activate peroxisome proliferator-activated receptor-*γ* (PPAR-*γ*) [37] in muscles, which might have beneficial effects on insulin sensitivity. However, the influences of M on insulin signaling in liver cells need further investigation.

Although M and B might promote glucose consumption and stimulate insulin sensitivity through different mechanisms, when they were conjugated in the compound of MB salt, significantly improved activities were obtained. Together with the results of gluconeogenesis, our findings demonstrated that MB salt had potent activities in modulating glucose metabolism in HepG2 cells which were superior to that of M or B alone.

When M and B were administered at the same time to treat HepG2 cells, they stimulated AMPK, reduced intracellular TG, promoted glucose consumption, and suppressed glucose production similar to the MB salt at an equal molar concentration (data not shown in this study). However, the* in vivo* situation may be different. When administered alone, M and B are poorly absorbed as single molecules [40, 41], which may have unfavourable influences on their pharmacological activities* in vivo*. However, it was found that MB salt could be easily dissolved in strong acids [24]. So,* in vivo* in the stomach, it is possible that MB salt can be fully dissolved in gastric acid. As a single molecule, it is possible that MB salt may have improved pharmacokinetic parameters as compared to M and B when administered together, which deserves further study.

In a previous report [42], M and B were simply mixed together at a molecular ratio of 1 : 1 and were used to treat diabetic mice. Although the mixture of M and B was shown to reduce blood glucose, there was no comparison between the mixture and M or B alone in that report [42]. Furthermore, that composition was proved to be unstable [24, 42], which might limit its potential clinical application. The MB salt is a single molecule; it is stable and can be easily stored [24], which may provide convenience for future clinical application.

M and B are natural compounds which belong to different classes and their chemical skeletons are distinct. As a result, they exhibit different toxicities in HepG2 cells and have different mechanisms in modulating metabolisms, as discussed above. As a single molecule, the MB salt contains an M group and a B group. It can be speculated that the mechanisms and pathways of MB salt in stimulating lipid and glucose metabolisms may have combined features as compared to those of M or B alone, as illustrated in Figure 10. Indeed, our results in the present study prove that MB salt stimulates TG metabolism and inhibits glucose production through the AMPK pathway. However, the upstream signals recruited by MB salt to activate AMPK still need further investigation. In addition, the influences of MB salt on glycolysis, glucose oxidation, membrane translocation of GLUTs, and the insulin signaling pathway are not clear; they need detailed investigation and comparison with M or B when administered alone.

In conclusion, the new compound of MB salt has potent activities in stimulating AMPK, reducing intracellular lipid accumulation, enhancing glucose consumption, and suppressing gluconeogenesis in HepG2 cells. Our results may support MB salt as a new kind of agent for the development of novel lipid or glucose-lowering drugs. Future studies should focus on animal experiments to validate the metabolism-modulating activities of MB salt* in vivo*.

## Figures and Tables

**Table 1:** Primers for real-time PCR (5′ to 3′).

Gene	Forward primer	Reverse primer
LDLR	aggacggctacagctaccc	ctccaggcagatgttcacg
PEPCK	gctctgaggaggagaatgg	tgctcttgggtgacgataac
G6Pase	gtgaattaccaagactcccag	gcccatggcatggccagaggg
SREBP1c	cgacatcgaagacatgcttcag	ggaaggcttcaagagaggagc
ChREBP	agagacaagatccgcctgaa	cttccagtagttccctcca
FAS	gacatcgtccattcgtttgtg	cggatcaccttcttgagctcc
SCD1	ggtgatgttccagaggaggta	ggcagagtagtcataggaaagg
GAPDH	agccacatcgctcagacac	gcccaatacgaccaaatcc
